# Formulation Development and In Vitro/In Vivo Characterization of Methotrexate-Loaded Nanoemulsion Gel Formulations for Enhanced Topical Delivery

**DOI:** 10.3390/gels9010003

**Published:** 2022-12-22

**Authors:** Muhammad Shahid Latif, Asif Nawaz, Mufarreh Asmari, Jalal Uddin, Hidayat Ullah, Saeed Ahmad

**Affiliations:** 1 Advanced Drug Delivery Lab, Gomal Centre of Pharmaceutical Sciences, Faculty of Pharmacy, Gomal University, Dera Ismail Khan 29050, Pakistan; 2 Department of Pharmaceutical Chemistry, College of Pharmacy, King Khalid University, Asir 61421, Saudi Arabia; 3 Institute of Chemical Sciences, Gomal University, Dera Ismail Khan 29050, Pakistan; 4 Institute of Biotechnology and Microbiology, Bacha Khan University, Charsadda 24460, Pakistan

**Keywords:** methotrexate, nanoemulsion gel, olive oil, almond oil, clove oil, chitosan, topical drug delivery

## Abstract

Methotrexate-loaded oil-in-water nanoemulsion formulations were prepared using the high shear homogenization technique. A drug excipient study (ATR-FTIR) was carried out to investigate the compatibility between the drug, the polymers, and its admixtures. The thermal stability of the nanoemulsion formulations was evaluated by subjecting them to a heating and cooling cycle. The prepared nanoemulsion formulations (FNE1 to FNE6) were evaluated for particle size, PDI value, and entrapment efficiency (EE). They were analyzed for morphological information using transmission electron microscopy. The drug (methotrexate)-loaded nanoemulsion formulations (FNE2, FNE4, and FNE6) were then converted into nanoemulsion gel formulations by adding 1% chitosan (polymer) as a gelling agent. The nanoemulsion gel formulations (FNEG2, FNEG4, and FNEG6) were investigated for physicochemical parameters, viscosity, spreadability, extrudability, drug content, and skin irritation. Various penetration enhancers (olive oil, clove, and almond oil) were employed to examine the potency of the prepared nanoemulsion gel formulations. In vitro drug release, ex vivo permeation, skin drug retention, and stability tests were carried out for evaluation of the prepared nanoemulsion gel formulations (FNEG2, FNEG4, and FNEG6). The data obtained from the in vitro study were subjected to the kinetic model, and the Korsemeyer–Peppas model was best fitted to the data. The nanoemulsion gel formulation FNEG6 showed the maximum controlled drug release and followed an anomalous, non-Fickian release mechanism. The use of almond oil in the preparation of the nanoemulsion gel formulation FNEG6 helped the penetration of the drug across stratum corneum and the restructuring of the properties of skin and resulted in a higher penetration and retention of methotrexate in a deeper layer of the skin. The current study concluded that the methotrexate-loaded nanoemulsion gel formulation FNEG6 showed the best optimum release, permeation, and retention results as compared to the available oral tablets’ formulations, followed by a low serum concentration and the maximum drug retention, which is beneficial in treating skin infections and reducing systemic toxicity.

## 1. Introduction

Topical drug delivery is also known as a skin drug-delivery system. Skin is the superficial and largest part of the body [1]. Topical drug delivery is used to treat various skin infections locally [2]. There are many advantages of topical drug-delivery systems, they offer better patient compliance, are easy to apply over a larger surface area, and avoid first pass metabolism and gastrointestinal irritations. Topical drug delivery is easy for application over the skin and offers quick therapy termination in the case of any side effects appearing. This system lowers the daily dose of medications and avoids fluctuations in drug plasma concentration [3].

However, topical drug delivery possess some disadvantages, such as causing skin irritation, contact dermatitis, rashes, itchiness, and low permeation of the drug across skin [4]. The outer layer of the skin is comprised of the stratum corneum, a rigid horny layer that protects our body against foreign invaders and is the main barrier against the penetration of the drug into a deeper layer of the skin [5]. Formulations such as noisome, liposomes, solid lipid nanoparticles, and nanostructured lipid carriers could be used as alternatives for the effective and safe use of medication. However, these formulations also have certain limitations [6].

Methotrexate is an anti-cancerous drug used for different types of autoimmune diseases, such as rheumatoid arthritis, psoriasis, and different types of cancers. Methotrexate possesses anti-inflammatory, immune-suppressive, and cytotoxic properties to produce therapeutic benefits [7]. The toxicity of methotrexate has been depicted in the renal, pulmonary, bone marrow, and liver systems [8]. The topical use of methotrexate is challenging, because methotrexate disassociates at the body’s pH.

Nanodermatology is a promising and significant field in the management and treatment of various skin infections [9]. The skin acts a first mechanical barrier against the transdermal application of medication. In order to enhance a drug’s permeation across the skin, various chemical enhancers can be used in the drug’s formulations. These enhancers interact with the skin components and facilitate the permeation of the drug across the stratum corneum layer of the skin [10]. Different studies have shown that the use of penetration enhancers enhanced skin permeation as well as retention across skin [11].

Olive oil is a liquid fat that is found in olives. It exhibited antioxidant and anti-inflammatory activities and is widely used in topical formulations for the management and treatment of various skin infections [12]. Olive oil increases dihydroquercetin distribution in the skin and is used as a penetration enhancer in topical formulations [13]. Almond oil is found in the dried kernels of almonds. It is used in the formulation of perfume, cosmetics, and aromatherapy. Almond oil is comprised of oleic and linoleic essential fatty acids, which contribute as a good penetration properties for the skin [14]. Clove oil belongs to essential oil and is found in clove trees. The concept behind the transdermal penetration enhancement of clove oil is that clove oil disrupts the structured properties of stratum corneum lipids with a concurrent increase in intercellular diffusion [15].

Nowadays, scientists have developed a greater attention toward developing nanoemulsion gel formulations for treating various skin infections [16]. Nanoemulsion gel formulations facilitate a drug’s release onto the skin by offering a drug reservoir that influences the drug to release from the inner phase to the outer phase [17]. The network polymer and crosslink density have a direct influence on the drug release [18]. The ability of the drug to permeate through the skin barrier (stratum corneum) and the drug’s affinity to diffuse out from its vehicle have a greater influence regarding the drug’s permeation over the skin [19]. Oily droplets from the gel network are released from the nanoemulsion gel formulations [20]. The oil droplets’ penetration into the skin barrier (stratum corneum) takes place. The drug will then be directly delivered without transfer via a nanoemulsion hydrophilic phase.

Nanoemulsion gel formulations exhibit higher viscosity as compared to nanoemulsion formulations and inhibit the absorption of the drug [21]. Nanoemulsion gel formulations offer maximum drug retention in the deeper layers of the skin and inhibit the absorption of the drug into the blood stream, allowing for more effectiveness in the deeper layers of the skin [22]. The present study was aimed at formulating a stable oil-in-water methotrexate-loaded nanoemulsion. One percent chitosan was used for converting the nanoemulsion into nanoemulsion gel formulations. The formulated nanoemulsion gel was formulated for a deeper dermal deposition of methotrexate for the local treatment of skin diseases.

## 2. Results and Discussion

### 2.1. ATR FTIR Study

**Figure 1:**
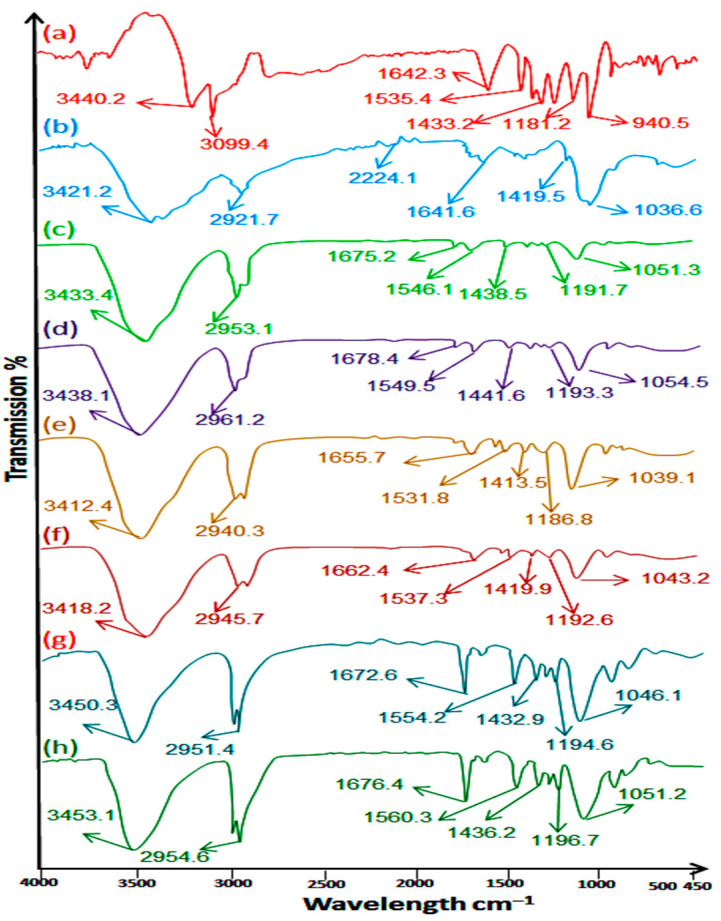
ATR FTIR spectra of (a) methotrexate, (b) chitosan, (c) FNE1, (d) FNE2, (e) FNE3, (f) FNE4, (g) FNE5, (h) FNE6.

An ATR-FTIR study of pure methotrexate, polymers, excipients, and methotrexate-loaded nanoemulsion formulations (FNE1-FNE6) was carried out for evaluating the purity of drug and to rule out any incompatibilities between the drug (MTX), the polymers, and the excipients used in the preparation of the MTX-loaded nanoemulsion formulations (FNE1-FNE6). The ATR-FTIR spectrum of methotrexate exhibited its characteristics bands at 3450 cm^−1^, which were attributed to the O–H stretched band from the carboxyl group, and a band appearing at 3080 cm^−1^ was attributed to the primary amine (N-H stretched band). Bands appearing in the range of 1600 cm^−1^ to 1670 cm^−1^ were attributed to the stretched carboxylic group (C=O), and the spectrum appearing at a range of 1500 cm^−1^ to 1550 cm^−1^ was attributed to the formation of an amide group [23] overlapping aromatic stretched spectra (–C=C). Bands appearing at a range of 1200 cm^−1^ to 1400 cm^−1^ were attributed to carboxylic groups’ stretching (–C–O). Bands appearing at 930 cm^−1^ were attributed to hydroxyl band (O–H), and the band appearing at 820 cm^−1^ was attributed to aromatic rings. The present study revealed that the drug (MTX), surfactant (PEG-400), co-surfactant (Tween-80), and natural oils (clove oil, almond oil and olive oil) used in the preparation of the nanoemulsion formulation showed no sort of incompatibilities or interactions. The prepared MTX-loaded nanoemulsion formulation (FNE1-FNE6) showed its original and characteristic peaks to be preserved, and no significant change was observed in the prepared formulation’s spectrum (Figure 1).

### 2.2. Thermodynamic Stability Analysis of Nanoemulsion Formulations

The prepared MTX-loaded nanoemulsion formulations (FNE1-FNE6) were evaluated for thermodynamic evaluation under stressed conditions. Different parameters (color, odor, phase separation, centrifugation, and thermodynamics) were evaluated for the prepared formulations. The present study depicted that the MTX-loaded nanoemulsion formulations passed the thermodynamic evaluation test and proved to be stable for further use (Table 1).

### 2.3. Organoleptic Analysis of Prepared Nanoemulsion

**Figure 2:**
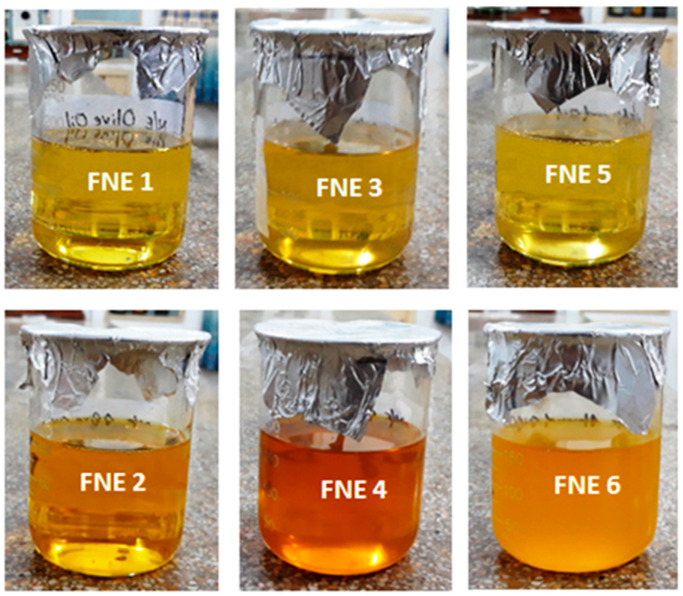
Organoleptic analysis of methotrexate-loaded nanoemulsion.

The prepared nanoemulsions were physically evaluated and found to be transparent and clear, with excellent homogeneity (Figure 2). The pH of the methotrexate-loaded nanoemulsion formulations ranged between 5.48 ± 0.41 and 5.72 ± 0.21. This is acceptable for application as a transdermal system. The pH values for the blank nanoemulsion formulations as well as for the methotrexate-loaded nanoemulsion formulations are expressed in Table 2. The pH exhibited by the prepared formulations (FNE1−FNE6) showed a value within the acceptable range of the skin (Table 2).

### 2.4. Particle Size, Polydispersity Index, and Zeta Potential

**Figure 3:**
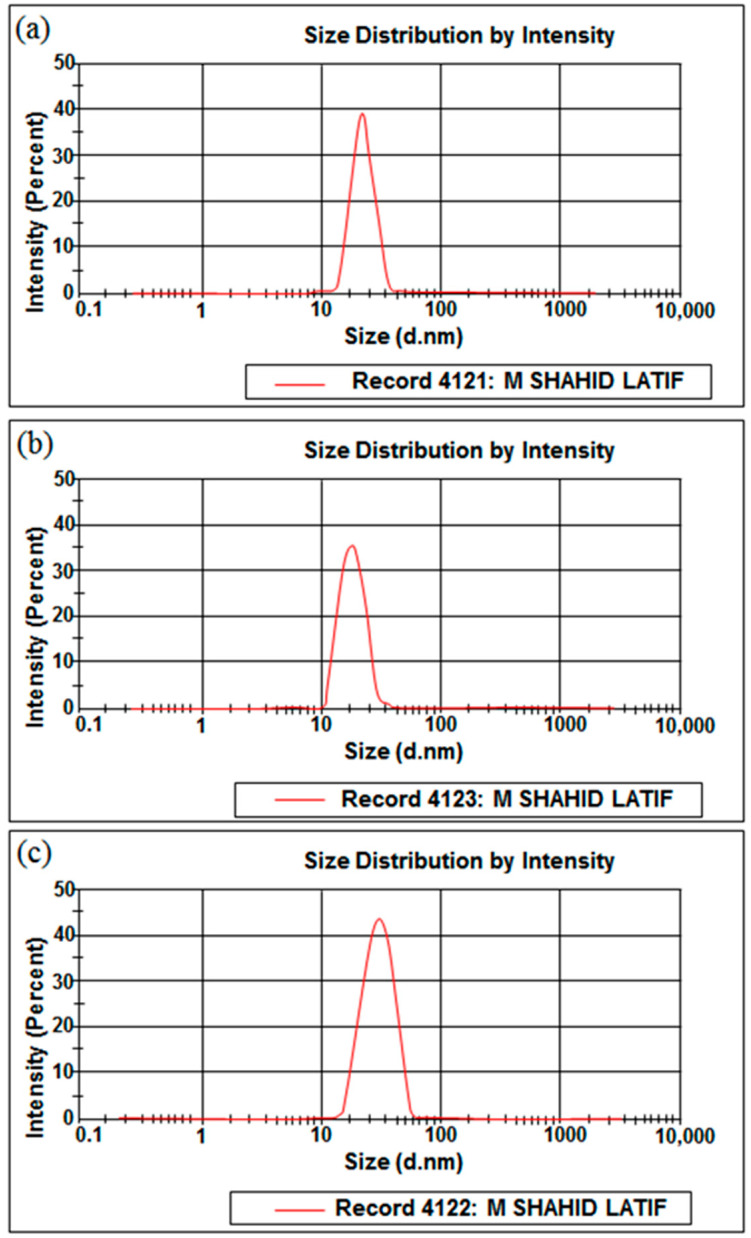
Zeta size of (**a**), FNE2, (**b**), FNE4, and (**c**) FNE6.

The prepared nanoemulsion formulations (FNE1–FNE6) exhibited a globule size ranging from 18.71 ± 3.45 nm to 28.58 ± 4.31 nm. The PDI value of the prepared nanoemulsion formulations ranged between 0.251 ± 0.04 and 0.732 ± 0.04. The zeta potential value of all formulated nanoemulsion formulations ranged between −8.59 ± 0.17 mV and −13.4 ± 0.22 mV (Table 3). The zeta size of MTX-loaded nanoemulsion formulations (FNE2, FNE4 and FNE6) has been showed in Figure 3.

### 2.5. Entrapment Efficiency and Drug Content Evaluation 

The prepared MTX-loaded nanoemulsion formulations (FNE1-FNE6) showed an average entrapment efficiency ranging between 75.38 ± 2.3% and 76.87 ± 1.9%. The drug solubility and compatibility with the oil phase and the excipients used in the preparation of the formulations have a great impact on the entrapment efficiency and drug content values. The present study depicted good agreement between the drug compatibility and the oil phase and the excipients used in the preparation of the MTX-loaded nanoemulsion formulations. The prepared MTX-loaded nanoemulsion formulations exhibited a percentage entrapment efficiency ranging between 75.38 ± 2.3 % and 78.12 ± 1.6 % and a percentage drug content value ranging between 86.1 ± 0.36 % and 91.5 ± 0.52 % (Table 3).

### 2.6. Transmission Electron Microscopy

**Figure 4:**
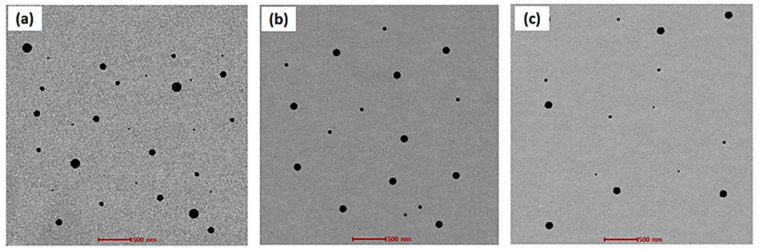
TEM images of the methotrexate-loaded nanoemulsion formulations: (**a**) FNE2, (**b**) FNE4, and (**c**) FNE6.

Morphological evaluation of the prepared MTX-loaded nanoemulsion formulations was carried out using transmission electron microscopy [24]. The TEM study depicted that the prepared MTX-loaded nanoemulsion formulations were dispersed uniformly. The shape of the prepared formulations was spherical and well-defined. The TEM study depicted that formulations FNE2, FNE4, and FNE6 showed an even and uniform distribution of the drug (MTX). The addition of natural oils (clove oil, almond oil, and olive oil) in the preparation of the MTX-loaded nanoemulsion formulation is in good agreement, because these natural oils have the best penetrative ability. They alter the barrier nature (stratum corneum) of the skin and allow for the maximum amount of the drug to penetrate into the deeper layer of the skin, whereby this deeper layer acts as reservoir for drug accumulation and treats the topical infection locally. The addition of chitosan with the MTX-loaded nanoemulsion gel formulations further produced a gel-layer structure and offered a controlled drug release from the formulations. Moreover, the spherical shape of the prepared formulations aids in squeezing from the pores of the skin, thereby promoting maximum skin permeability. The TEM images of the prepared MTX-loaded nanoemulsion formulations are shown below in Figure 4a–c.

### 2.7. Preparation of Methotrexate Nanoemulsion Gel

Nanoemulsion gel formulations were clear, bio-adhesive, and non-irritating formulations. The prepared MTX-loaded nanoemulsion formulations were converted into nanoemulsion gel formulations using 1% chitosan (*w*/*w*) as a gel-forming base [25]. 

### 2.8. Characterization of MTX-Loaded Nanoemulsion Gel Formulations 

#### 2.8.1. pH, Rheological Behavior, and Assay of Gel Formulations

The prepared nanoemulsion gel formulations showed pH values of FNEG2; 5.42 ± 0.25, FNEG4; 5.61 ± 0.31 and FNEG6; 5.53 ± 0.42, respectively (Table 4). The pH values of the prepared hydrogel formulations were in accordance with the skin’s pH. The pH values of the prepared formulations were best suited for topical application without causing any sort of skin irritation [26]. Viscosity is a crucial factor in the preparation of transdermal drug delivery. The viscosity of the topical preparation has a direct impact on drug release, spreadability, stability, and ease of application over the body [27]. The use of polymers and excipients used in the formulations of topical applications also influences their viscosity. The viscosity of the nanoemulsion gel formulations FNEG2, FNEG4, and FNEG6 showed an insignificant difference; this is because the amount of chitosan was kept constant in all the formulations (*p* > 0.05). However, a slight increase in the viscosity of FNEG2 was observed; this might be due to the higher viscosity of the oil (olive oil) used in the formulation. The prepared nanoemulsion gel formulations exhibited viscosities of FNEG2; 9986 ± 13.5, FNEG4; 9843 ± 12.3 and FNEG6; 9812 ± 13.1 cps, respectively, at a constant shear speed (40 rpm). Non-Newtonian pseudoplastic flow behavior was observed in the prepared nanoemulsion gel formulations, and this is advantageous in topical preparations for maximum coverage when applied over the skin [28].

#### 2.8.2. Spreadability

The therapeutic efficacy of the topical preparation depends on its spreadability. The spread of the topical application over the skin surface is known as spreadability. The optimum spreadability facilitates the topical preparation in coming out of its container with small shear stress. The topical formulation’s spreadability is influenced by low and high temperatures. The spreadability of the topical preparations increases with an increase in temperature, and the viscosity deceases with a decrease in temperature. The prepared methotrexate-loaded nanoemulsion gel formulations showed spreadability values of FNEG2; 9986 ± 13.5, FNEG4; 9843 ± 12.3 and FNEG6; 21.46 ± 1.65 g cm/s, respectively (Table 4). The prepared formulations showed an insignificant difference (*p* > 0.05). This might be due to the fact that the amounts of surfactant and co-surfactant used in the formulation of the nanoemulsion gels were kept constant. 

#### 2.8.3. Extrudability and Drug Content

The extrusion of the prepared topical application from the tube is known as extrudability. The esomeprazole-loaded nanoemulsion gel formulations exhibited an acceptable ability to extrude out from the tube. They showed extrudability values of FNEG2; 88.27 ± 0.54, FNEG4; 85.43 ± 0.34 and FNEG6; 87.15 ± 0.27, respectively (Table 4). The results were in an acceptable range. The prepared formulations were homogeneous in nature, clear, and showed a high drug content of FNEG2; 93.12 ± 0.45%, FNEG4; 95.36 ± 0.52% and FNEG6; 94.61 ± 0.63%, respectively (Table 4).

### 2.9. Skin Irritation Test 

**Figure 5:**
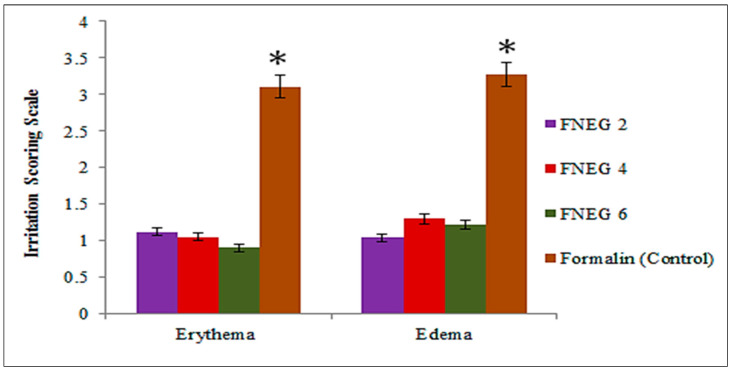
Skin irritation studies: erythema and edema. Data expressed as mean ± SD; *n* = 3. One-way ANOVA followed by post hoc Tukey test; * *p* < 0.05).

The skin irritation potential study was carried out for all prepared methotrexate-loaded nanoemulsion gel formulations (Table 4). The formulations did not cause any symptoms of inflammation, swelling, or any other changes on the skin. Hence, the study indicated that the methotrexate-loaded nanoemulsion gel formulations were nonirritating and can be used for topical applications (Figure 5).

### 2.10. In Vitro Drug Release Study

**Figure 6:**
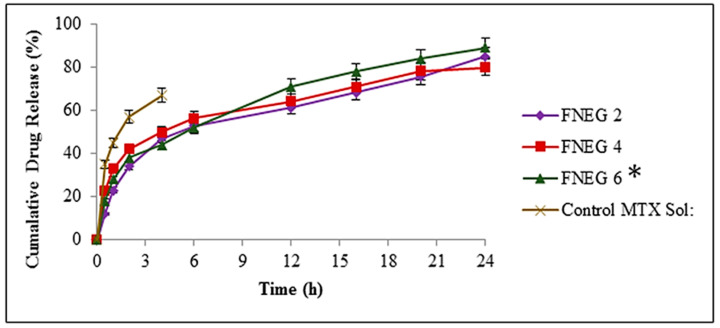
In vitro drug release of methotrexate-loaded nanoemulsion gel formulations. Data are expressed as mean ± SD; *n* = 3. One-way ANOVA followed by post hoc Tukey test (*p* < 0.05), FNEG2, FNEG4, and FNEG6 (* *p* < 0.05).

This study was carried out to evaluate the drug release profile of prepared nanoemulsion gel formulations. The aliquots were analyzed by UV visible spectrophotometer at λ_max_ 303 nm. A Franz diffusion cell was used for evaluating the in vitro drug release behavior of the prepared formulations. The receptor medium was filled with freshly prepared phosphate buffer solution (pH 5.5) in a simulation of the skin pH. The temperature of the receptor medium was kept at 32 ± 0.5 °C. Tuffryn membrane (diameter 2.5 mm and pore size 0.45 µm) was placed in between the donor and receptor compartment. The methotrexate-loaded nanoemulsion gel formulations FNEG2, FNEG4, and FNEG6 were placed in the donor compartment, and methotrexate solution was also placed in the donor compartment and served as the control group. In the first two hours, the prepared gel formulations exhibited a burst drug release. The burst release within the initial 2 h is useful, as in the case of treating skin infections. The prepared nanoemulsion gel formulations showed releases in descending order: FNEG6 (89.72 ± 1.3%) > FNEG2 (85.61 ± 1.4%) > FNEG4 (80.72 ± 1.5%) (ANOVA, *p* < 0.05). The drug release is regulated by the interactions of the drug, the surfactants, and the drug partitioning between the aqueous and oil phases. The nanoemulsion gel formulations must attain a smaller globule size for achieving the maximum amount of drug release. The prepared nanoemulsion gel formulations showed drug release in a regulated manner. They exhibited a reliable, effective, and simple approach for attaining a controlled drug release (Figure 6).

### 2.11. Drug Release Kinetics

A different kinetic model was used for investigating the release behavior of the prepared nanoemulsion gel formulations, and the Korsmeyer–Peppas model was best fitted to the data (Table 5). A linear equation was exhibited by the formulated gel formulations. An anomalous, non-Fickian release mechanism was observed for the methotrexate-loaded nanoemulsion gel formulations. The value of ‘n’ obtained ranged between 0.583 and 0.732. 

### 2.12. In Vitro Skin Permeation of Methotrexate-loaded Nanoemulsion Gel Formulations

**Figure 7:**
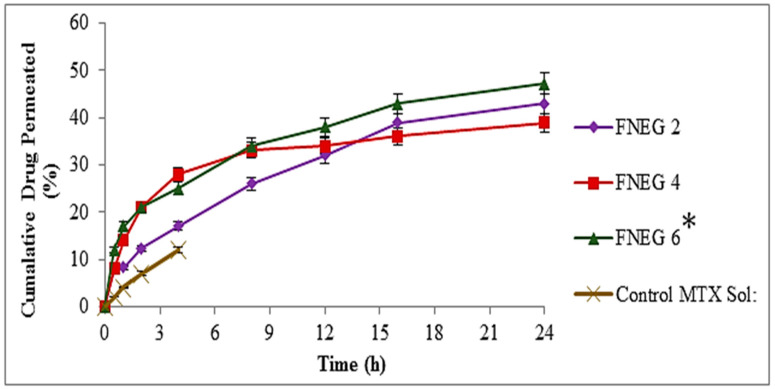
Ex vivo permeation of methotrexate-loaded nanoemulsion gel formulations. Data are expressed as mean ± SD; *n* = 3. One-way ANOVA followed by post hoc Tukey test (*p* < 0.05), FNEG 2, FNEG 4, and FNEG 6 (* *p* < 0.05).

In vitro permeation studies were carried out for evaluating the skin permeation of the methotrexate-loaded nanoemulsion gel formulations. The prepared nanoemulsion gel formulations were evaluated for in vitro skin permeation using a Franz diffusion cell. Methotrexate solution was used as a control group. The skin permeation evaluation of the prepared nanoemulsion gel formulation is depicted in Figure 7. Permeation profiles were used to evaluate the total amount of methotrexate that penetrated the skin after 24 h, as well as the permeation flux and enhancement ratio. After 24 h, the cumulative quantity of methotrexate that permeated from the prepared nanoemulsion gel formulations and methotrexate solution (control) was evaluated. The permeation value of FNEG6 (47.37 ± 3.1 µg/cm^2^) was substantially greater than that which penetrated from the FNEG2 (43.21 ± 3.3 µg/cm^2^) and FNEG4 (39.42 ± 3.5 µg/cm^2^) (ANOVA, *p* < 0.01), respectively, whereas the methotrexate solution (control) showed the least permeation up to 4 h. Furthermore, the formulation FNEG6 (2.124 ± 0.34 µg/cm^2^/h) showed the maximum flux value as compared to FNEG2 (1.971 ± 0.21 µg/cm^2^/h) and FNEG4 (2.016 ± 0.28 µg/cm^2^/h). The order of improvement in skin permeation was FNEG6 > FNEG2 > FNEG4. Furthermore, the addition of natural oils (olive oil, clove oil, and almond oil) to the formulations also effects the permeation of methotrexate from the prepared nanoemulsion gel formulations. Among all the formulations, FNEG6, containing almond oil, exhibited the maximum amount of methotrexate, since almond oil is renowned for its rich concentrations of oleic and linoleic essential fatty acids, which help to give it unequalled penetrative and restructuring properties and result in enhanced skin penetration. The methotrexate drug belongs to BCS class IV drugs, and the concentration of oleic acid was found to be a good enhancer for these drugs for transdermal application. 

### 2.13. Drug Retention Analysis

**Figure 8:**
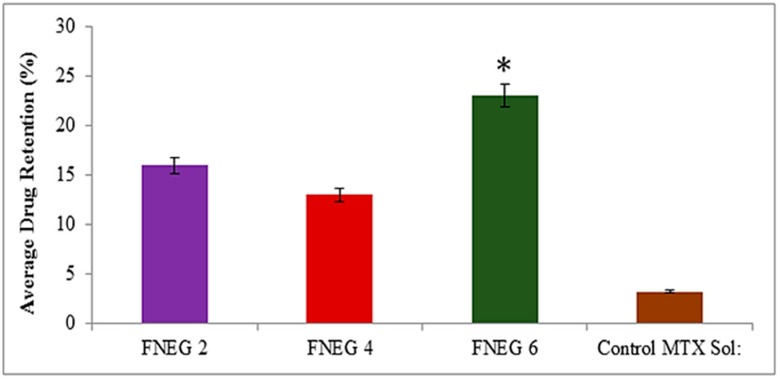
Amount of drug retained (μg/cm^2^) in dermal layer of skin (* *p <* 0.01 vs. FNEG 2, FNEG 4, and FNEG 6).

The methotrexate-loaded nanoemulsion gel formulations were investigated for methotrexate retention in the deeper layer of the skin. The study showed that formulation FNEG6 (23.10 ± 1.32 µg/cm^2^) showed an increased drug retention value as compared to FNEG2 (16.21 ± 1.54 µg/cm^2^) and FNEG4 (13.29 ± 1.61 µg/cm^2^), whereas the methotrexate solution (control) showed the minimum amount of drug retention (3.2 ± 1.21 µg/cm^2^) (ANOVA, *p* < 0.01). The reason for this might be due to the presence of natural oils (olive oil, clove oil, and almond oil) in the preparation of the nanoemulsion gel formulations. The study revealed that FNEG6 showed the maximum amount of drug retention in the dermis layer of the skin. The reason for this might be due to the presence of almond oil, which is comprised of oleic and linoleic essential fatty acids. The almond oil alters the barrier nature of the stratum corneum and facilitates the methotrexate (BCS class IV) drug in breeching the stratum corneum, resulting in the maximum accumulation of drug in the deeper layer of the skin (Figure 8).

### 2.14. In Vivo Studies

In vivo studies were carried out for the prepared nanoemulsion gel formulations (FNEG2, FNEG4, and FNEG6). They were evaluated for a serum comparative bioavailability study. The prepared nanoemulsion gel formulations were investigated for the various parameters Cmax, tmax, AUC, t^1/2^, kel, and MRT (Table 6). They were evaluated for time and mean plasma concentration, as shown in Table 6, and on the basis of MRT, AUC_0-t,_ and biological half-life values, the methotrexate-loaded nanoemulsion gel formulations were ranked as FNEG6 > FNEG4 > FNEG2. The FNEG6 showed 9.1 ± 0.21(µg/mL) plasma levels, as compared to FNEG4 (8.7 ± 0.34 µg/mL) and FNEG2 (8.1 ± 0.13 µg/mL), respectively (Table 6). The prepared nanoemulsion gel formulations are advantageous for various skin diseases. Methotrexate, when used topically, showed the minimum drug permeation and the maximum drug retention in the deeper layer of the skin. This accumulation of drug in the deeper layer is beneficial in treating skin infection locally. The current study showed low plasma residual concentrations from the prepared nanoemulsion gel formulations’ results in minimizing systemic side effects. The calculated values showed that the biological half-life (t^1/2^) of the methotrexate drug in rabbits increased from 4–10 h^−1^ and ranged between 14.9 ± 1.98 to 15.8 ± 1.78. As a result, the drug delivered by the nanoemulsion gel formulation will remain for a prolonged period of time.

**Figure 9:**
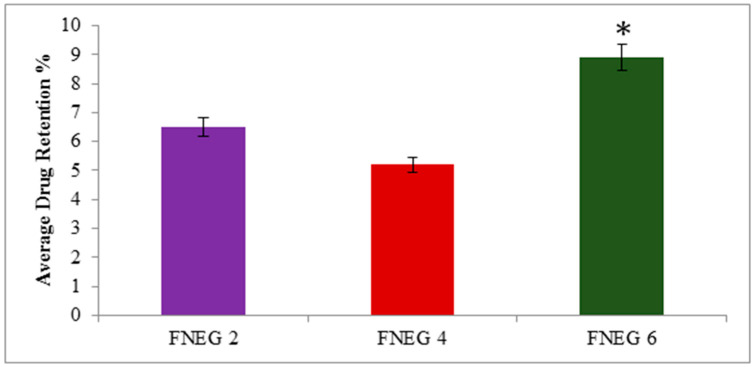
Methotrexate concentrations in the dermal layer of skin from methotrexate-loaded nanoemulsion gel formulations (FNEG2, FNEG4, and FNEG6), respectively, calculated from in vivo studies (* *p* < 0.001).

The methotrexate-loaded nanoemulsion gel formulations showed a lower elimination rate constant: FNEG6 (0.031 ± 0.002 h^−1^) < FNEG4 (0.036 ± 0.003 h^−1^) < FNEG2 (0.040 ± 0.005 h^−1^) and a prolonged mean residence time: FNEG6 (9.1 ± 0.21 h) > FNEG4 (8.7 ± 0.34 h) > FNEG6 (8.1 ± 0.13 h), both of which support the drug’s extended activity from the nanoemulsion gel formulations. The increased bioavailability of the medications is also shown by the increased AUC value attained with the nanoemulsion gel formulations FNEG6 (173.8 ± 21.7 µg/mL·h) > FNEG4 (165.5 ± 19.6 µg/mL·h) > FNEG2 (159.2 ± 18.2 µg/mL·h). The reason for this might be attributed to the hepatic first pass effect being bypassed and stomach degradation being avoided. The study depicted that FNEG6 showed the best in vivo results, and it can be used as a suitable candidate for treating skin diseases topically (Figure 9).

### 2.15. Stability Determination

The prepared nanoemulsion gel formulations were kept for stability tests. The formulation (FNEG6) was evaluated for pH, PDI, surface charge, and changes in droplet size (Table 7). There was no change in the clarity of the nanoemulsion gel formulation, and no phase separation was observed after 60 days of storage in the stated conditions. The stability study depicted that the prepared nanoemulsion gel formulations were physically and chemically stable. There was no change observed in the pH of the prepared nanoemulsion gel formulation; however, a slight increase in the globule size was observed. This slight increase might be due to the presence of the gelling agent (chitosan) and the increased kinetic energy of the globules that might lead to random globule collision and aggregation. The methotrexate-loaded nanoemulsion gels formulations passed the stability test, which showed that this formulation is stable at the stated temperatures and is safe to preserve for a longer period of time.

## 3. Conclusions

This current study depicted that the prepared nanoemulsion gel formulations were the most suitable candidates used topically. In topical administration therapy, the stratum corneum is the main challenging barrier. The methotrexate-loaded nanoemulsion gel formulations containing various natural oils were used as penetration enhancers. The prepared nanoemulsion gel formulation FNEG6 showed the best physicochemical properties. The in vitro release study of formulation FNEG6 showed the maximum controlled drug release. The use of almond oil in the preparation of the nanoemulsion gel formulation FNEG6 helps in the penetration of the drug across the stratum corneum and in restructuring the properties of skin, resulting in a higher penetration and the retention of methotrexate in the deeper layer of the skin. The prepared nanoemulsion gel formulations depicted the best optimum release, permeation, and retention results as compared to the available oral tablets’ formulations. The prepared nanoemulsion gel formulations depicted low serum concentrations and the maximum drug retention, which is beneficial in treating skin infections and reducing systemic toxicity. 

## 4. Materials and Methods

### 4.1. Materials

Methotrexate (Sigma-Aldrich, Inc., St. Louis, MO, USA, +1-314-771-5765) was used as the model drug. Tween-80 and PEG 400 (Dow Chemical Company, 693 Washington St #627, Midland, MI 48640, USA) were used in the preparation of the nanoemulsion gels. Chitosan (MW:3000), and triethanolamine (Sigma-Aldrich, Inc., St. Louis, MO, USA, +1-314-771-5765) were used as gelling agents in the preparation of the nanoemulsion gels. Olive oil, clove oil, and almond oil (Marhaba Industries, Lahore, Pakistan) were used as penetration enhancers in the nanoemulsion gels. The chemicals used in the preparation of nanoemulsion gel formulations were of analytical grade. 

### 4.2. Preparation of Methotrexate O/W Nanoemulsion

The high shear homogenization method was carried out for the preparation of the methotrexate-loaded oil-in-water nanoemulsion. The formulation was comprised of two phases, i.e., an oil phase comprised of olive oil, clove oil, and almond oil, and an aqueous phase comprised of methotrexate, tween 80, PEG 400, and distilled water. The oil phase and the aqueous phase were placed in a water bath for 30 min at 70 °C. The nanoemulsion was prepared by subjecting the oil phase drop wise into the aqueous phase. The final preparations were placed on a magnetic stirrer until a clear and uniform emulsion was observed. In order to remove entrapped air bubbles, the prepared emulsion was placed in a sonicator for 10 min. The clear emulsion was carried out to a high shear homogenization (Daihan, Yongin, Republic of Korea) at 10,000 rpm for 15 min. The same process was carried out for all nanoemulsion formulations (Table 8) [29].

### 4.3. ATR FTIR Study

The drug, polymers, and excipients were observed in an ATR-FTIR analysis. The obtained ATR-FTIR analysis of the drug, polymers, and excipients, as well as the admixtures (FNE1-FNE6), was evaluated for possible incompatibilities. The ingredients used in the preparation of the formulations (FNE1-FNE6) were placed over the diamond crystal of an FTIR machine, and the knob of the apparatus was pressed. A total scan resolution of 4000 and 600 cm^−1^ was used for spectrum evaluation [30].

### 4.4. Thermodynamic Stability Analysis of Optimized Nanoemulsion Formulations

The thermodynamic stability analysis of the methotrexate-loaded nanoemulsion formulations was carried out by placing them room temperature and storing at −20 °C for 24 h. The prepared formulations returned to their original state within a time period of just 2–3 min, showing the better stability of the formulations. Triplicate readings were obtained as mean ± SD [31]. Centrifugation of the prepared nanoemulsion formulations was carried out at 10,000 rpm for 10 min. Phase separation and turbidity were evaluated in the prepared nanoemulsion formulations (FNE1-FNE6) [32]. The process of a heating and cooling cycle was also carried out for the prepared nanoemulsion formulations. For determination of the cooling stability of the formulations, the refrigerator was set at a temperature of 4 °C, and for the heating cycle, the temperature was set at 45 °C [33]. The thermodynamic stability study was conducted for 48 h, and results were obtained as mean ± SD. 

### 4.5. Characterization of Prepared Methotrexate Nanoemulsion Formulations

#### 4.5.1. Size, Polydispersity Index, and Zeta Potential 

The particle size and particle size distribution of the prepared nanoemulsion formulations were evaluated using a zeta sizer Nano ZS 90 (Malvern Instruments; Worcestershire, UK). Uniform dispersion was obtained by mixing 10 μL of the prepared nanoemulsion formulation and deionized water to make the final volume of 5 mL. Triplicate readings for the diluted formulations were obtained at a temperature of 25 ± 0.1 °C [34].

#### 4.5.2. Physicochemical Assessment of Nanoemulsion Preparations 

A calibrated pH meter (InoLab^®^, Bremen, Germany) was used for the determination of the pH values of the prepared nanoemulsion formulations. The results were obtained as mean ± SD [35]. A UV visible spectrophotometer (UV- 1601, SHIMADZU, Kyoto, Japan) was used for evaluating the drug content of the prepared nanoemulsion formulations (FNE1-FNE6). An aliquot of 2 mL was obtained from the formulations and was poured into an eppendorf tube. The eppendorf tube was kept for centrifugation at 10,000 rpm for 10 min. After proper centrifugation, the collected supernatant was obtained and diluted with phosphate buffer solution (pH 7.4). The collected sample was placed over a magnetic stirrer for 10 min. A UV visible spectrophotometer was used for evaluation of the collected samples at λ_max_ of 303 nm. One mL of methanol and sediment was incorporated to extract the entrapped methotrexate in the prepared nanoemulsion formulations, and phosphate buffer (pH 7.4) was used for dilution of the obtained aliquots. A UV visible spectrophotometer was used for the evaluation of absorbance at λ_max_ of 303 nm. The supernatant and sediment drug loading were used for evaluation of the drug content [36].

Drug content for the prepared formulations was evaluated using the following equation:

Drug Content = Drug in supernatant + Drug in sediment(1)

#### 4.5.3. Entrapment Efficiency (EE)

Centrifugation was carried out for the prepared nanoemulsion formulations (FNE1-FNE6). The temperature was set at 20 °C to obtain separation between the nanoemulsion formulations. A UV visible spectrophotometer was used for evaluation of the separated filtrate at λ_max_ 303 nm. Phosphate buffer (pH 7.4) was used for the construction of the standard curve of methotrexate. The results were collected as mean ± SD. The blank nanoemulsion formulations were used as control formulations. The amount of the drug (methotrexate) initially added to the nanoemulsion formulations was subtracted from the free drug (methotrexate) in the filtrate.

The following equation was used for the evaluation of entrapment efficiency [37]:

Drug EE = [(Added Drug − Free Drug)/(Drug added)] × 100(2)

#### 4.5.4. Nanoemulsion Transmission Electron Microscopy

TEM (TECNAI G^2^ (200 kV) HR-TEM from the FEI company, Hillsboro, Oregon, United States) was carried out for investigating the structure of the nanoemulsion formulations. The prepared nanoemulsion formulations were centrifuged at 12,000 rpm for 5 min, and the aqueous phase was separated. The sediments were mixed with a few drops of osmium tetra oxide (OTO) and fixed at 8 °C for 2 h. An amount of 0.1 M phosphate buffer was used for a washing medium. Acetone was added for dehydration of the samples. The collected sample was kept over carbon film (400 mesh), and images were magnified at 2500× at an accelerated voltage of 8.0 KV [38].

### 4.6. Preparation of Methotrexate Nanoemulsion Gel

Water-soluble chitosan (MW: 3000) was used for the preparation of the nanoemulsion gel formulations. One percent (*w*/*w*) chitosan gel solution was used to convert the nanoemulsion formulations to nanoemulsion gel formulations. The chitosan gel was prepared by dispersing 1 g of chitosan into 100 mL distal water containing 1% acetic acid. The prepared gel was kept overnight before incorporating into the nanoemulsion formulations. A few drops of triethanolamine were also added to the dispersion to produce the nanoemulsion gel formulations and to neutralize the pH of the prepared nanoemulsion gel formulations [39]. The formulations were placed in a sonicator for the removal of entrapped air. Table 9 demonstrates the compositions of the MTX-loaded nanoemulsion gel formulations.

### 4.7. Characterization of Nanoemulsion Gel Formulations

#### 4.7.1. Organoleptic Appearance and Homogeneity 

The clarity, appearance, and homogeneity of the methotrexate-loaded nanoemulsion gel formulations were evaluated visually. A small quantity of the nanoemulsion gel formulations was taken in between the thumb and index finger for evaluating homogeneity [23].

#### 4.7.2. pH and Rheological Study

The pH of the prepared formulations was evaluated using a calibrated pH meter (InoLab^®^, Bremen, Germany). The pH meter was calibrated using standard buffer solution, and 1 g of the prepared gel formulations was incorporated into 25 mL of distilled water [40]. A Brookfield viscometer was used for the evaluation of the rheological characteristics of the prepared gel formulations. A spindle no. 64 was used for evaluating the viscosity of the prepared nanoemulsion gel formulations [41].

#### 4.7.3. Spreadability

A drag and slip apparatus was used for evaluation of the spreadability of the prepared nanoemulsion gel formulations. This apparatus was made of wood containing a pulley. It was comprised of two glass slides: one glass was fixed, and the other glass was allowed to move. A weighted amount (2 g) of the prepared nanoemulsion gel formulation was placed in between the glass slides, and a measured amount of weight (50 g) was placed on the upper glass slide. The time for the upper glass to cover a distance of 8 cm was noted [42]. Triplicate results were observed as mean ± SD. 

The prepared nanoemulsion formulations were evaluated for spreadability using the following equation:

S = M × L/T(3)

where S represents the spreadability of the nanoemulsion gel formulation, M represents the weight of the upper glass slide, L represents the glass slide’s length, and T represents the total time required to reach a distance of 8 cm.

#### 4.7.4. Extrudability

Aluminum collapsible tubes were used for evaluating the extrudability of the prepared nanoemulsion formulations. The aluminum collapsible tubes were filled with 20 g of the prepared gel formulation. The caps of the tubes were removed, and the tubes were gently pressed. The prepared gel formulations were squeezed out of the tubes until the pressure dissipated. Triplicate results were observed as mean ± SD [43].

The extrudability of the prepared nanoemulsion formulations was evaluated using the following formula:

Extrudability = Weight applied to extrude gel/Area (cm^2^) (4)

#### 4.7.5. Drug Content

The drug content was evaluated by placing 1 g of the prepared nanoemulsion gel formulation into 100 mL of phosphate buffer solution. A UV visible spectrophotometer (UV- 1601, SHIMADZU, Kyoto, Japan) was used for evaluating the drug content of the prepared nanoemulsion gel formulations at a wavelength of 303 nm [44].

### 4.8. Skin Irritation Test

This test was performed for all the prepared nanoemulsion gel formulations. The NOC was approved by the Gomal Centre of Pharmaceutical Science, Faculty of Pharmacy, Gomal University, Dera Ismail Khan, KP, Pakistan. Male healthy albino rabbits weighing 2–2.5 kg were used in this study. The rabbits were provided with the standard recommended food [45]. They were kept at room temperature (25 ± 2 °C). Relative humidity was controlled at 50 ± 10%. The rabbits were divided into 3 groups. Group 1 was left untreated and served as the control group. Group 2 rabbits were treated with the prepared nanoemulsion gel formulations, and group 3 rabbits were treated with a standard irritant formalin. The skin irritation score was performed visually. Skin irritation was evaluated as follows: “0” represents no skin irritation, “1” represents slight skin irritation, “2” represents well-defined skin irritation, “3” represents moderate skin irritation, and “4” represents scar formations [46].

### 4.9. In Vitro Release and Kinteic Profiling 

The in vitro release behavior of the prepared nanoemulsion gel formulations was evaluated using a Franz diffusion cell apparatus (Perme-Gear, Hellertown, PA, USA). The partitioning of the donor and receptor compartment was carried out using a Tuffryn membrane (artificial membrane) with a pore size 0.45 µm. Fresh phosphate buffer (pH 5.5) was used in the receptor compartment as a simulation of skin pH. The temperature was maintained at 32 ± 2 °C as a simulation to skin temperature. From the receptor compartment, 1 mL sample was collected, and 1 mL fresh buffer solution (pH 5.5) was added to the receptor compartment in order to maintain a sink condition. The collected samples were analyzed at λ_max_ 303 using a UV visible spectrophotometer. Triplicate results were observed as mean ± SD. 

The data obtained from the in vitro release study were fitted in a power law equation:

Mt/M∞ = k t^n^ (5)

where Mt/M∞ represents the release of the drug, k represents the power constant, and n represents the exponent of diffusion. The n value showed drug transport behavior [31].

### 4.10. Ex Vivo Permeation

This study was performed in the Advance Drug Delivery Lab (ADDL), Gomal Centre of Pharmaceutical Science (GCPS), Faculty of Pharmacy, Gomal University, Dera Ismail Khan, KP, Pakistan. Male healthy albino rabbits of 2–2.5 kg weight were used in this study. The rabbits were fed with the standard recommended food [24] and kept at room temperature (25 ± 2 °C). Relative humidity was controlled at 50 ± 10%. An overdose of a ketamine and xylazine injection was used to sacrifice rabbits, and then the skin of the rabbits was surgically excised. The adhered fats from the skin were removed by placing the skin in warm water for 10 to 15 s. The excised skin was cleaned with 0.9% sodium chloride solution and placed at −20 ± 1 °C until further use [24].

A Franz diffusion cell apparatus was used for investigating the ex vivo permeation behavior of the methotrexate from the prepared nanoemulsion gel formulations. Fresh phosphate buffer (pH 7.4) was used in the receptor compartment that was simulated to blood pH. The temperature of the compartments was maintained at 37 ± 1 °C, in a simulation of body temperature. Magnetic stirrer beads were rotated over fixed 100 rpm. The methotrexate-loaded nanoemulsion gel formulation (500 mg) was placed in the donor compartment, and excised skin was placed in between the donor and the receptor compartment. An aliquot of 2ml was obtained from the receptor compartment, and freshly prepared buffer solution (pH 7.4) was replaced in the receptor compartment to maintain the sink condition. After pre-determined time intervals, the collected aliquots were sprectrophotometrically evaluated using a UV visible spectrophotometer at a 303 nm wavelength [47].

### 4.11. Skin Drug Retention Analysis

After the completion of the ex vivo permeation study, the prepared nanoemulsion gel formulations were observed for skin drug retention analysis. The skin was removed carefully from Franz diffusion cells. The skin was cleaned with phosphate buffer solution, dried, and cut into small pieces. It was kept in phosphate buffer solution (pH 7.4) and stirred for 24 h. Methanol was added for the extraction of the retained skin drug. The samples were centrifuged at 10,000 rpm for 10 min. The collected aliquots were evaluated spectrophotometrically at λ_max_ 303 nm. The results were obtained in triplicate as mean ± SD [48].

### 4.12. In Vivo Studies

For in vivo study, proper approval was given from GCPS, Faculty of Pharmacy, Gomal University, Dera Ismail Khan, KP, Pakistan. Male healthy rabbits weighing 2–2.5 kg were used for the in vivo studies. The rabbits selected for the in vivo study were given the standard food and were maintained at room temperature with relative humidity [31]. An injected solution of ketamine and xylazine was used as anesthesia. After proper anesthetization, the backs of the rabbits were shaved with the help of an electric trimmer. The rabbis were categorized into three various groups. Each group consisted of 3 rabbits. The prepared methotrexate-loaded nanoemulsion gel formulation (FNEG 2) was applied to Group A; the formulation (FNEG 4) was applied to group B; and the formulation (FNEG 6) was applied to Group C, respectively. From all the rabbits, a 0.5 mL blood sample was collected. Methanol was added to the blood samples, and centrifugation was carried out at 10,000 rpm for 5 min. The HPLC method was employed for evaluating drug plasma content. The supernatant was filtered and dissolved in 0.5 mL the HPLC mobile phase pH 6 (0.2 M Na_2_HPO_4_ and 0.2 M citric acid; 2:1) and acetonitrile in 90:10 *v*/*v*. The rabbits were sacrificed by injecting them with an overdose of ketamine and xylazine (i.m). The obtained skin was cleaned and washed with 0.9% sodium chloride solution. The obtained skin was cut into small pieces. The skin was further placed in distilled water for 24 h. The obtained filtrate was analyzed for residual drug concentrations using HPLC [49].

### 4.13. Stability Studies

The prepared nanoemulsion formulations were subjected for stability tests at various conditions. The stability studies were carried out for a time period of 60 days. The formulations were placed in a glass container in the refrigerator at 4 ± 1 °C and at an accelerated temperature 40 ± 2 °C. After pre-determined time intervals on day 0, 7, 14, 28, and 60, the formulations were evaluated for physical changes, clarity, color, phase separation, particle size, pH, zeta potential, drug content, and poly-dispersity [50].

### 4.14. Statistical Analysis

Statistical analysis was carried out using a student’s t-test or ANOVA. A *p*-value < 0.05 was considered significant, and all the tests were evaluated in triplicate and interpreted as mean ± SD, (*n* = 3). SPSS software version-18 was used for analyzing statistical data (SPSS Inc., Chicago, IL, USA).

## Figures and Tables

**Table 1:** Thermodynamics stability analysis of nanoemulsion formulations (FNE1-FNE6).

F.Code	Characteristics									
	Color		Odor		Phase Separation		Centrifugation		Thermodynamics	
	4 ± 2 °C	40 ± 2 °C	4 ± 2 °C	40 ± 2 °C	4 ± 2 °C	40 ± 2 °C	4 ± 2 °C	40 ± 2 °C	4 ± 2 °C	40 ± 2 °C
FNE1	Pale yellow	Pale yellow	No change	No change	No	No	Stable	Stable	Passed	Passed
FNE2	Pale yellow	Pale yellow	No change	No change	No	No	Stable	Stable	Passed	Passed
FNE3	Pale yellow	Pale yellow	No change	No change	No	No	Stable	Stable	Passed	Passed
FNE4	Dark brown	Dark brown	No change	No change	No	No	Stable	Stable	Passed	Passed
FNE5	Pale yellow	Pale yellow	No change	No change	No	No	Stable	Stable	Passed	Passed
FNE6	Light brown	Light brown	No change	No change	No	No	Stable	Stable	Passed	Passed

**Table 2:** Organoleptic analysis of prepared nanoemulsion formulations (FNE1–FNE6).

Parameters	FNE1	FNE2	FNE3	FNE4	FNE5	FNE6
Physical Appearance	Transparent	Transparent	Transparent	Transparent	Transparent	Transparent
Clarity	Clear	Clear	Clear	Clear	Clear	Clear
pH	5.48 ± 0.41	5.72 ± 0.21	5.51 ± 0.32	5.63 ± 0.25	5.59 ± 0.13	5.64 ± 0.44
Homogeneity	Excellent	Excellent	Excellent	Excellent	Excellent	Excellent

Data expressed as mean ± SD, (*n* = 3).

**Table 3:** Physicochemical characteristics of methotrexate nanoemulsions.

F.Code	Particle Size	PDI	Zeta Potential (mV)	Entrapment Efficiency (%)	Drug Content (%)
FNE1	18.71 ± 3.45	0.386 ± 0.03	−11.6 ± 0.15	0.0	0.0
FNE2	20.81 ± 2.87	0.617 ± 0.01	−9.33 ± 0.24	76.87 ± 1.9	87.2 ± 0.71
FNE3	28.83 ± 4.23	0.732 ± 0.04	−13.4 ± 0.22	0.0	0.0
FNE4	17.52 ± 5.13	0.391 ± 0.02	−8.59 ± 0.17	75.38 ± 2.3	86.1 ± 0.36
FNE5	16.76 ± 2.48	0.251 ± 0.04	−13.2 ± 0.43	0.0	0.0
FNE6	28.58 ± 4.31	0.699 ± 0.03	−9.44 ± 0.52	78.12 ± 1.6	91.5 ± 0.52

Data expressed as mean ± SD, (*n* = 3).

**Table 4:** Physicochemical characteristics of methotrexate nanoemulsion gel formulations.

F. Code	pH	Viscosity (Centipoise)	Spreadability (g cm/s)	Extrudability	Drug Content (%)	Skin Irritation
FNEG2	5.42 ± 0.25	9986 ± 13.5	20.13 ± 1.23	88.27 ± 0.54	93.12 ± 0.45	Nil
FNEG4	5.61 ± 0.31	9843 ± 12.3	20.82 ± 1.45	85.43 ± 0.34	95.36 ± 0.52	Nil
FNEG6	5.53 ± 0.42	9812 ± 13.1	21.46 ± 1.65	87.15 ± 0.27	94.61 ± 0.63	Nil

Data were expressed as mean ± SD, (*n* = 3).

**Table 5:** Kinetic profiling of methotrexate-loaded nanoemulsion gel formulations.

F.Code	Korsmeyer Model			
	K ± SD	R^2^	n	Release Mechanism
FNEG2	1.874 ± 1.039	0.963	0.732	Non Fickian Diffusion
FNEG4	1.561 ± 2.861	0.921	0.646	Non Fickian Diffusion
FNEG6	2.571 ± 0.005	0.944	0.583	Non Fickian Diffusion

**Table 6:** Pharmacokinetic parameters of methotrexate from methotrexate-loaded nanoemulsion gel formulations upon administration to skin in rabbits up to 24 h.

Parameters	FNEG2	FNEG4	FNEG6
Cmax (µg/mL)	8.1 ± 0.13	8.7 ± 0.34	9.1 ± 0.21
Tmax (h)	12	12	12
K (h^−1^)	0.040 ± 0.005	0.036 ± 0.003	0.031 ± 0.002
t ½	14.9 ± 1.98	15.4 ± 2.14	15.8 ± 1.78
AUC 0–t (µg/mL.h)	159.2 ± 18.2	165.5 ± 19.6	173.8± 21.7
MRT (h)	11.69 ± 0.26	11.82 ± 0.19	12.10 ± 0.32

Cmax, peak plasma concentration of drug; Tmax, time at which Cmax was observed; K, elimination rate constant; t ^½^, elimination half-life; AUC 0–t, area under the plasma concentration time curve from 0 h to time t; MRT, mean residence time.

**Table 7:** Storage stability of methotrexate nanoemulsion gel formulation (FNEG6) at refrigerated and accelerated temperatures for 60 days.

Parameters	Temperature	
	4 ± 2 °C	40 ± 2 °C
Particle size	24.76 ± 4.12	24.21 ± 3.98
PDI	0.29 ± 0.35	0.27 ± 0.19
Zeta potential (mV)	−3.81 ± 4.21	−3.86 ± 3.29
pH	6.14 ± 0.32	6.31 ± 0.16
Phase separation	Nil	Nil
Clarity	Transparent and clear	Transparent and clear
Drug content (%) ± SD	97.21 ± 0.32	97.10 ± 0.12
Color change	No change	No change

Data expressed as mean ± SD (*n* = 3).

**Table 8:** Composition of 0.25% w/w methotrexate nanoemulsion formulation (FNE1-FNE6).

F. Code	Water Phase				Oil Phase		
	Drug MTX (g)	Tween 80 (g)	PEG 400 (g)	Distilled Water	Olive Oil (g)	Clove Oil (g)	Almond Oil (g)
FNE1	0.0	5	5	Q.S	7.5	0.0	0.0
FNE2	0.25	5	5	Q.S	7.5	0.0	0.0
FNE3	0.0	5	5	Q.S	0.0	7.5	0.0
FNE4	0.25	5	5	Q.S	0.0	7.5	0.0
FNE5	0.0	5	5	Q.S	0.0	0.0	7.5
FNE6	0.25	5	5	Q.S	0.0	0.0	7.5

**Table 9:** Preparation of methotrexate-loaded nanoemulsion gel formulations (*w*/*w*, %).

F. Code	Prepared MTX Nanoemulsion	Chitosan	Triethanolamine	Distilled Water
FNEG2	50	1	1	48
FNEG4	50	1	1	48
FNEG6	50	1	1	48

## Data Availability

Not applicable.

## References

[1] Latif M.S., Al-Harbi F.F., Nawaz A., Rashid S.A., Farid A., Al Mohaini M., Alsalman A.J., Al Hawaj M.A., Alhashem Y.N. (2022). Formulation and Evaluation of Hydrophilic Polymer Based Methotrexate Patches: In Vitro and In Vivo Characterization. Polymers.

[2] Sabbagh F., Kim B.S. (2021). Recent advances in polymeric transdermal drug delivery systems. J. Control. Release.

[3] Ramadon D., McCrudden M.T.C., Courtenay A.J., Donnelly R.F. (2021). Enhancement strategies for transdermal drug delivery systems: Current trends and applications. Drug Deliv. Transl. Res..

[4] Das Kurmi B., Tekchandani P., Paliwal R., Paliwal S.R. (2017). Transdermal Drug Delivery: Opportunities and Challenges for Controlled Delivery of Therapeutic Agents Using Nanocarriers. Curr. Drug Metab..

[5] Ghosalkar S., Singh P., Ravikumar P. (2021). Emerging topical drug delivery approaches for the treatment of Atopic dermatitis. J. Cosmet. Dermatol..

[6] Chakole C.M., Chauhan M.K. (2021). Research progress of nanostructured lipid carriers in ocular drug delivery. Drug Deliv. Lett..

[7] Shang H., Younas A., Zhang N. (2022). Recent advances on transdermal delivery systems for the treatment of arthritic injuries: From classical treatment to nanomedicines. Wiley Interdiscip. Rev. Nanomed. Nanobiotechnol..

[8] Srivastava S., Rasool M. (2022). Underpinning IL-6 biology and emphasizing selective JAK blockade as the potential alternate therapeutic intervention for rheumatoid arthritis. Life Sci..

[9] Zeien J., Qiu W., Triay M., Dhaibar H.A., Cruz-Topete D., Cornett E.M., Urits I., Viswanath O., Kaye A.D. (2021). Clinical implications of chemotherapeutic agent organ toxicity on perioperative care. Biomed. Pharmacother..

[10] Damiani G., Pacifico A., Linder D.M., Pigatto P.D., Conic R., Grada A., Bragazzi N.L. (2019). Nanodermatology-based solutions for psoriasis: State-of-the art and future prospects. Dermatol. Ther..

[11] Gushiken L., Beserra F., Bastos J., Jackson C., Pellizzon C. (2021). Cutaneous Wound Healing: An Update from Physiopathology to Current Therapies. Life.

[12] Alhasso B., Ghori M.U., Conway B.R. (2022). Systematic Review on the Effectiveness of Essential and Carrier Oils as Skin Penetration Enhancers in Pharmaceutical Formulations. Sci. Pharm..

[13] Nunes A., Gonçalves L., Marto J., Martins A., Silva A., Pinto P., Martins M., Fraga C., Ribeiro H. (2021). Investigations of Olive Oil Industry By-Products Extracts with Potential Skin Benefits in Topical Formulations. Pharmaceutics.

[14] Shalaby K. (2022). Effect of Olive Oil Acidity on Skin Delivery of Diclofenac: In vitro Evaluation and ex vivo Skin Permeability Studies. J. Biomed. Nanotechnol..

[15] Moore E.M., Wagner C., Komarnytsky S. (2020). The Enigma of Bioactivity and Toxicity of Botanical Oils for Skin Care. Front. Pharmacol..

[16] Teaima M.H., Alsofany J.M., El-Nabarawi M.A. (2022). Clove Oil Endorsed Transdermal Flux of Dronedarone Hydrochloride Loaded Bilosomal Nanogel: Factorial Design, In vitro Evaluation and Ex vivo Permeation. AAPS PharmSciTech.

[17] Souto E.B., Cano A., Martins-Gomes C., Coutinho T.E., Zielińska A., Silva A.M. (2022). Microemulsions and Nanoemulsions in Skin Drug Delivery. Bioengineering.

[18] Ojha B., Jain V.K., Gupta S., Talegaonkar S., Jain K. (2021). Nanoemulgel: A promising novel formulation for treatment of skin ailments. Polym. Bull..

[19] Parekh K., A Mehta T., Dhas N., Kumar P., Popat A. (2021). Emerging Nanomedicines for the Treatment of Atopic Dermatitis. AAPS PharmSciTech.

[20] Harshitha V., Swamy M.V., Kumar D.P., Rani K.S., Trinath A. (2020). Nanoemulgel: A process promising in drug delivery system. Res. J. Pharm. Dos. Technol..

[21] Saidi M., Dabbaghi A., Rahmani S. (2019). Swelling and drug delivery kinetics of click-synthesized hydrogels based on various combinations of PEG and star-shaped PCL: Influence of network parameters on swelling and release behavior. Polym. Bull..

[22] Tungadi R., Susanty W., Wicita P., Pido E. (2018). Transdermal Delivery of Snakehead Fish (Ophiocephalus striatus) Nanoemulgel Containing Hydrophobic Powder for Burn Wound. Pharm. Sci..

[23] Khatoon K., Ali A., Ahmad F.J., Hafeez Z., Rizvi M., Akhter S., Beg S. (2021). Novel nanoemulsion gel containing triple natural bio-actives combination of curcumin, thymoquinone, and resveratrol improves psoriasis therapy: In vitro and in vivo studies. Drug Deliv. Transl. Res..

[24] Kassem A.A., Salama A., Mohsen A.M. (2022). Formulation and optimization of cationic nanoemulsions for enhanced ocular delivery of dorzolamide hydrochloride using Box-Behnken design: In vitro and in vivo assessments. J. Drug Deliv. Sci. Technol..

[25] Patel D., Patel B., Thakkar H. (2021). Lipid Based Nanocarriers: Promising Drug Delivery System for Topical Application. Eur. J. Lipid Sci. Technol..

[26] Zeb A., Qureshi O.S., Yu C.-H., Akram M., Kim H.-S., Kim M.-S., Kang J.-H., Majid A., Chang S.-Y., Bae O.-N. (2017). Enhanced anti-rheumatic activity of methotrexate-entrapped ultradeformable liposomal gel in adjuvant-induced arthritis rat model. Int. J. Pharm..

[27] Rai V.K., Mishra N., Yadav K.S., Yadav N.P. (2018). Nanoemulsion as pharmaceutical carrier for dermal and transdermal drug delivery: Formulation development, stability issues, basic considerations and applications. J. Control. Release.

[28] Zahid F., Batool S., Ud-Din F., Ali Z., Nabi M., Khan S., Salman O., Khan G.M. (2022). Antileishmanial Agents Co-loaded in Transfersomes with Enhanced Macrophage Uptake and Reduced Toxicity. AAPS PharmSciTech.

[29] Jyothi V.G.S., Ghouse S.M., Khatri D.K., Nanduri S., Singh S.B., Madan J. (2022). Lipid nanoparticles in topical dermal drug delivery: Does chemistry of lipid persuade skin penetration?. J. Drug Deliv. Sci. Technol..

[30] Aleanizy F.S., Taha E.I., Salem-Bekhit M.M., Felimban A.M.J., Al-Suwayeh S.A., Al-Joufi F.A., Muharram M.M., Alqahtani F.Y., Shakeel F., Youssof A.M.E. (2021). Formulation and in vitro and in vivo evaluation of surfactant-stabilized mucoadhesive nanogels for vaginal delivery of fluconazole. Drug Dev. Ind. Pharm..

[31] Rajitha P., Shammika P., Aiswarya S., Gopikrishnan A., Jayakumar R., Sabitha M. (2019). Chaulmoogra oil based methotrexate loaded topical nanoemulsion for the treatment of psoriasis. J. Drug Deliv. Sci. Technol..

[32] Karadurmus L., Corman M.E., Uzun L., Ozkan S.A. (2022). Enantioselective recognition of esomeprazole with a molecularly imprinted sol–gel-based electrochemical sensor. Mikrochim. Acta.

[33] Rashid S.A., Bashir S., Naseem F., Farid A., Rather I.A., Hakeem K.R. (2021). Olive Oil Based Methotrexate Loaded Topical Nanoemulsion Gel for the Treatment of Imiquimod Induced Psoriasis-like Skin Inflammation in an Animal Model. Biology.

[34] Mahajan R., Tandon R., Kalia A., Mahajan B.V.C. (2021). Nanoemulsion Formulation of *Ocimum gratissimum* Essential Oil and Its Antifungal Activity Against *Penicillium digitatum*. J. Nanosci. Nanotechnol..

[35] Abedinpour N., Ghanbariasad A., Taghinezhad A., Osanloo M. (2021). Preparation of Nanoemulsions of Mentha piperita Essential Oil and Investigation of Their Cytotoxic Effect on Human Breast Cancer Lines. Bionanoscience.

[36] Chrastina A., Welsh J., Borgström P., Baron V.T. (2022). Propylene Glycol Caprylate-Based Nanoemulsion Formulation of Plumbagin: Development and Characterization of Anticancer Activity. BioMed Res. Int..

[37] Mohammed N.K., Muhialdin B.J., Hussin A.S.M. (2020). Characterization of nanoemulsion of *Nigella sativa* oil and its application in ice cream. Food Sci. Nutr..

[38] Rashid S.A., Bashir S., Ullah H., ullah Shah K., Khan D.H., Shah P.A., Danish M.Z., Khan M.H., Mahmood S., Sohaib M. (2021). Development, characterization and optimization of methotrexate-olive oil nano-emulsion for topical application. Pak. J. Pharm. Sci..

[39] Nawaz A., Latif M.S., Alnuwaiser M.A., Ullah S., Iqbal M., Alfatama M., Lim V. (2022). Synthesis and Characterization of Chitosan-Decorated Nanoemulsion Gel of 5-Fluorouracil for Topical Delivery. Gels.

[40] Poonia N., Lather V., Kaur B., Kirthanashri S.V., Pandita D. (2020). Optimization and Development of Methotrexate- and Resveratrol-Loaded Nanoemulsion Formulation Using Box–Behnken Design for Rheumatoid Arthritis. ASSAY Drug Dev. Technol..

[41] Khan M.K., Khan B.A., Uzair B., Niaz S.I., Khan H., Hosny K.M., Menaa F. (2021). Development of Chitosan-Based Nanoemulsion Gel Containing Microbial Secondary Metabolite with Effective Antifungal Activity: In vitro and in vivo Characterizations. Int. J. Nanomed..

[42] Rani K.R.V., Rajan S., Bhupathyraaj M., Priya R.K., Halligudi N., Al-Ghazali M.A., Sridhar S.B., Shareef J., Thomas S., Desai S.M. (2022). The Effect of Polymers on Drug Release Kinetics in Nanoemulsion In Situ Gel Formulation. Polymers.

[43] Sevinç-Özakar R., Seyret E., Özakar E., Adıgüzel M.C. (2022). Nanoemulsion-Based Hydrogels and Organogels Containing Propolis and Dexpanthenol: Preparation, Characterization, and Comparative Evaluation of Stability, Antimicrobial, and Cytotoxic Properties. Gels.

[44] Burki I.K., Khan M.K., Khan B.A., Uzair B., Braga V.A., Jamil Q.A. (2020). Formulation Development, Characterization, and Evaluation of a Novel Dexibuprofen-Capsaicin Skin Emulgel with Improved In Vivo Anti-inflammatory and Analgesic Effects. AAPS PharmSciTech.

[45] Mulleria S.S., Marina K., Ghetia S.M. (2021). Formulation, Optimization and in vitro Evaluation of Apremilast Nanoemulgel for Topical Delivery. Int. J. Pharm. Investig..

[46] Nawaz A., Farid A., Safdar M., Latif M.S., Ghazanfar S., Akhtar N., Al Jaouni S.K., Selim S., Khan M.W. (2022). Formulation Development and Ex-Vivo Permeability of Curcumin Hydrogels under the Influence of Natural Chemical Enhancers. Gels.

[47] Valizadeh A., Shirzad M., Pourmand M.R., Farahmandfar M., Sereshti H., Amani A. (2020). Levofloxacin nanoemulsion gel has a powerful healing effect on infected wound in streptozotocin-induced diabetic rats. Drug Deliv. Transl. Res..

[48] Eze C.C., Ekeke N., Alphonsus C., Lehman L., Chukwu J.N., Nwafor C.C., Stillwaggon E., Meka A.O., Sawers L., Ikebudu J. (2021). Effectiveness of self-care interventions for integrated morbidity management of skin neglected tropical diseases in Anambra State, Nigeria. BMC Public Health.

[49] Latif M.S., Nawaz A., Rashid S.A., Akhlaq M., Iqbal A., Khan M.J., Khan M.S., Lim V., Alfatama M. (2022). Formulation of Polymers-Based Methotrexate Patches and Investigation of the Effect of Various Penetration Enhancers: In Vitro, Ex Vivo and In Vivo Characterization. Polymers.

[50] Alam Shah M.K., Azad A.K., Nawaz A., Ullah S., Latif M.S., Rahman H., Alsharif K.F., Alzahrani K.J., El-Kott A.F., Albrakati A. (2021). Formulation Development, Characterization and Antifungal Evaluation of Chitosan NPs for Topical Delivery of Voriconazole In Vitro and Ex Vivo. Polymers.

